# Rotamer-Resolved Vibronic and Cationic Properties of *m*-Aminostyrene: A Combined 2C-REMPI, Hole-Burning, and MATI Study

**DOI:** 10.3390/molecules31111866

**Published:** 2026-05-29

**Authors:** Rui Wang, Xiateng Qin, Keke Zhang, Yan Zhao, Changyong Li, Suotang Jia

**Affiliations:** 1 State Key Laboratory of Quantum Optics Technologies and Devices, Institute of Laser Spectroscopy, Shanxi University, Taiyuan 030006, China; 19861241857@163.com (R.W.); 18734558738@163.com (X.Q.); zhangkeke323@163.com (K.Z.); 2 Department of Physics and Electronics Engineering, Jinzhong University, Jinzhong 030619, China; 3 Collaborative Innovation Center of Extreme Optics, Shanxi University, Taiyuan 030006, China

**Keywords:** *m*-aminostyrene, rotamer, REMPI, hole-burning, MATI, vibronic spectrum, cation spectrum, vibrational assignment

## Abstract

*m*-Aminostyrene (MAS) is a key molecular scaffold with an electron-donating amino group and a conjugating vinyl group, exhibiting significant potential in photonic materials and biological applications due to its rotamerism and photoinduced behavior. Despite its importance, a comprehensive, rotamer-resolved investigation of its vibronic and cationic spectroscopic properties is lacking. Here, we report a high-resolution study on the *cis* and *trans* rotamers of jet-cooled MAS using two-color resonant enhanced multi-photon ionization (2C-REMPI), UV-UV hole-burning (HB), and mass-analyzed threshold ionization (MATI) spectroscopies, combined with density functional theory (DFT) calculations. The HB technique unambiguously resolves the vibronic spectra of each rotamer, overcoming the limitations of previous one-color REMPI studies. The excitation energies (S_1_ ← S_0_) are determined to be 30,416 cm^−1^ (*cis*) and 30,932 cm^−1^ (*trans*). The MATI spectra yield precise adiabatic ionization energies (AIEs) of 61,569 cm^−1^ (*cis*) and 61,274 cm^−1^ (*trans*). A comprehensive assignment of vibrational modes in both the S_1_ and D_0_ states is provided, revealing distinct mode activities and frequency shifts between the two rotamers. A propensity for Δν = 0 upon ionization is observed, indicating high geometrical similarity between the S_1_ and D_0_ states. This work provides a crucial spectroscopic blueprint for understanding the electronic and vibrational structure of MAS rotamers, with implications for the design of functionalized styrene-based molecular systems.

## 1. Introduction

Aminostyrenes, as push–pull aromatic systems combining an electron-donating amino group (-NH_2_) with a conjugating vinyl group (-CH=CH_2_), have attracted considerable interest due to their unique photophysical properties and potential applications in nonlinear optics, molecular electronics, and as monomers for functional polymers [1,2,3]. The isomeric position of the substituents on the benzene ring dramatically alters the electronic structure and intramolecular interactions. Specifically, meta-aminostyrene (MAS) presents a fascinating case where two distinct rotational isomers (rotamers), *cis* and *trans*, are expected to coexist. These rotamers can exhibit different spectroscopic signatures and reactivity, which is crucial for understanding biochemical processes and designing molecular switches [4].

Resonance-enhanced multiphoton ionization (REMPI) spectroscopy is a powerful tool for investigating the vibronic structure of electronically excited states (S_1_) of jet-cooled molecules, offering high sensitivity and spectral resolution [5,6,7,8]. Furthermore, mass-analyzed threshold ionization (MATI) spectroscopy provides a route to obtain high-resolution spectra of molecular cations, yielding precise adiabatic ionization energies (AIEs) and detailed information on the vibrational structure of the ionic ground state (D_0_) [9,10,11,12,13,14,15,16]. A combination of these techniques with UV-UV hole-burning (HB) spectroscopy allows for the unambiguous separation and identification of spectral features originating from different conformers or rotamers co-existing in a molecular beam [17,18,19,20,21,22,23].

Extensive studies have been reported for fluorinated anilines [24,25,26,27,28,29,30,31,32], fluorinated styrenes [33,34,35], methoxystyrenes [36,37], ethynylanilines [38], chloro-substituted styrenes [39,40,41], and methyl-substituted aromatic systems [42,43,44,45,46,47] using REMPI–MATI methods. The S_1_ excited state and D_0_ cationic state of *p*-aminostyrene have been thoroughly investigated by the Zeng group [48]. However, a complete, rotamer-resolved spectroscopic study covering both the S_1_ excited state and D_0_ cationic state of *m*-aminostyrene is very limited. The initial spectroscopic work on MAS was reported by Dong et al. [49] using one-color REMPI (1C-R2PI). While this study successfully identified the band origins of the *cis* and *trans* rotamers and provided a preliminary vibrational assignment, it suffered from a critical limitation. Specifically, the one-color scheme failed to record the complete spectrum for the *cis* rotamer because its S_1_ ← S_0_ transition energy is lower than its ionization threshold from the S_1_ state, leading to missing low-frequency vibrational information. Furthermore, a robust, rotamer-resolved analysis employing HB spectroscopy has been lacking.

In this work, we overcome these challenges by employing a combination of two-color REMPI (2C-REMPI), 3-color UV-UV HB, and MATI spectroscopies, supported by high-level DFT calculations. The 2C-REMPI scheme ensures the full detection of the S_1_ state vibronic features for both rotamers. The HB technique provides an unambiguous assignment of all spectral features to either the *cis* or *trans* rotamer. Finally, MATI spectroscopy is utilized to, for the first time, record the high-resolution cation spectra of both rotamers. This comprehensive study allows us to precisely determine the S_1_ ← S_0_ excitation energies and adiabatic IEs for both *cis* and *trans* rotamers of MAS. We provide detailed assignments of the vibrational modes in the S_1_ and D_0_ states.

## 2. Results

### 2.1. Rotamer-Resolved Vibronic Spectra by 2C-REMPI and Hole-Burning

**Figure 1:**
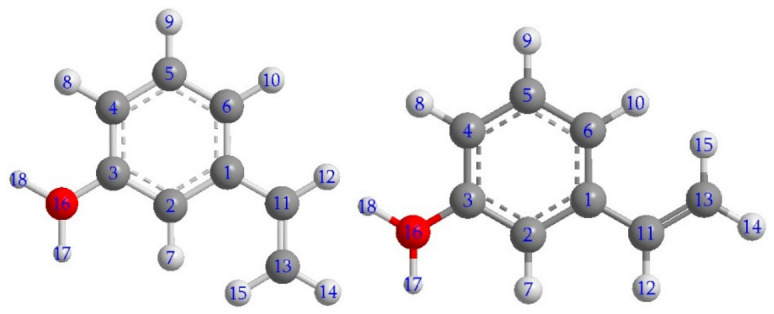
Molecular structures with atom labeling for *cis* and *trans* rotamers of *m*-aminostyrene.

**Figure 2:**
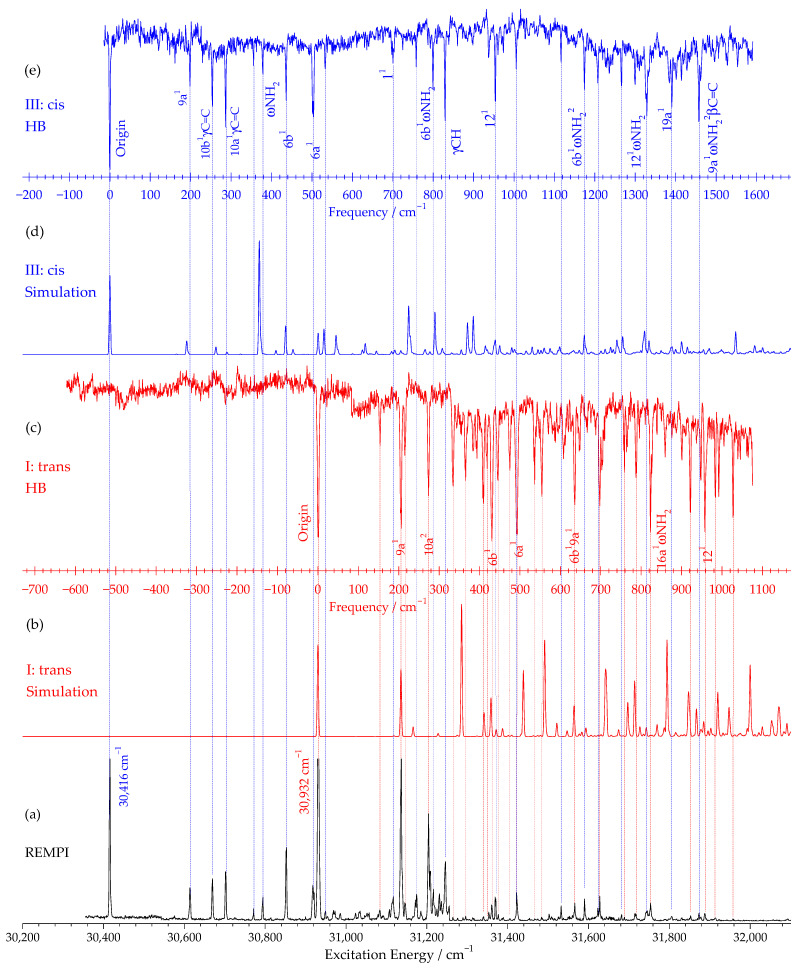
The two-color REMPI spectrum of *m*-aminostyrene (**a**); the Franck–Condon simulated spectra of the S_1_ ← S_0_ 0_0_^0^ transition for the *trans* (**b**) and *cis* (**d**) rotamers; the hole-burning (HB) spectra with the probe laser fixed at 30,416 cm^−1^ (*cis*, (**c**)) and 30,932 cm^−1^ (*trans*, (**e**)).

Theoretical calculations indicate that *m*-aminostyrene exists in two stable rotamers, *cis* and *trans*. The molecular geometries of these conformers are shown in Figure 1. The 2C-REMPI spectrum of MAS in the region of the S_1_ ← S_0_ electronic transition is shown in Figure 2. The spectrum exhibits a rich vibronic structure. The most intense band at 30,416 cm^−1^ is designated as the origin (0^0^) of the *cis* rotamer, while the band at 30,932 cm^−1^ is the origin (0^0^) of the *trans* rotamer. This assignment is based on UV-UV hole-burning (HB) spectroscopy: fixing the probe laser on either origin produced a distinct hole-burning spectrum, unambiguously separating the spectral features of the two rotamers.

This origin assignment is consistent with the behavior observed in *m*-methoxystyrene [37]. That system contains four conformers due to the independent orientations of the methoxy and vinyl groups. Regardless of the orientation of the methoxy group, the *cis* orientation of the vinyl group consistently exhibits a lower S_1_ excitation energy than the *trans* orientation. Specifically, when the methoxy group adopts the *cis* orientation, the excitation energies of the vinyl-*cis* (conformer II) and vinyl-*trans* (conformer IV) are 32,907 and 33,281 cm^−1^, respectively; when the methoxy group adopts the *trans* orientation, the excitation energies of the vinyl-*cis* (conformer I) and vinyl-*trans* (conformer III) are 32,767 and 33,222 cm^−1^, respectively. Importantly, this assignment of the S_1_ origins is further corroborated by the ionization-energy-based assignment, which relies on both calculated and measured IEs (see Section 3).

The HB spectra demonstrate that all significant bands in the 2C-REMPI spectrum can be assigned to either the *cis* or *trans* rotamer. Critically, the 2C-REMPI method, by using a fixed ionization laser at sufficiently high energy, successfully captures the complete vibronic spectrum for both rotamers, unlike the previous 1C-R2PI study [49] which was hampered by the ionization efficiency issues for the *cis* rotamer. The observed transitions and their assignments are summarized in Table 1 (*cis*) and Table 2 (*trans*).

**Figure 3:**
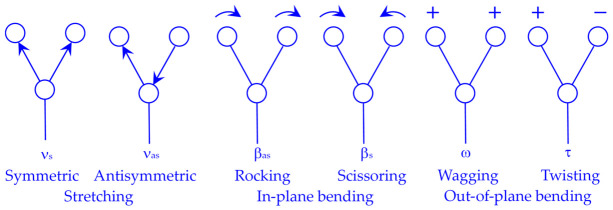
Six normal modes of the amino and methylene groups.

*m*-Aminostyrene contains two substituents, an amino group and a vinyl group, and consists of 18 atoms, giving a total of 48 normal vibrational modes. The normal modes localized on the benzene ring are named following the Wilson notation [50,51,52], totaling 30 modes. The normal modes localized on the substituents are denoted using Greek letters [51,52]: ν for stretching vibrations; β for in-plane bending vibrations; γ for out-of-plane vibrations; ω for wagging vibrations of the amino or methylene groups [53]; and τ for twisting vibrations [53]. The subscripts ‘s’ and ‘as’ denote symmetric and antisymmetric vibrations, respectively [51,52]. The amino and methylene groups each possess six normal modes, as illustrated in Figure 3. For the vinyl group, its two components, C_α_H and C=C, each exhibit three vibrational modes: in-plane bending, stretching, and out-of-plane vibrations.

Prominent active modes for both rotamers in the S_1_ state include low-frequency ring deformation modes (e.g., 10b, 9a, 6b, 6a), the ring breathing mode (1), and vibrations involving the substituents (e.g., βC=C, ωNH_2_). The calculated frequencies at the B3LYP/aug-cc-pVTZ level (scaled) are in good agreement with the experimental values, supporting our assignments.

For benzene derivatives, the S_1_ ← S_0_ transition typically involves expansion of the aromatic ring, predominantly activating in-plane vibrational modes [54,55,56]. However, unlike many other benzene derivatives, the REMPI spectra of *m*-aminostyrene exhibit a significant number of out-of-plane vibrations, including 10a, 10b, 7b, γC=C, γCH, and ωNH_2_, as can be seen from Table 1 and Table 2. Notably, the NH_2_ wagging mode (ωNH_2_), which is perpendicular to the ring plane, appears seven times in Table 1 and five times in Table 2. This observation is attributed to the substantial structural change accompanying the electronic transition: the amino group, which tilts out of the ring plane by approximately 24° in the ground state (see Tables S1 and S2), becomes nearly coplanar with the ring in the S_1_ state.

### 2.2. MATI Spectra

**Figure 4:**
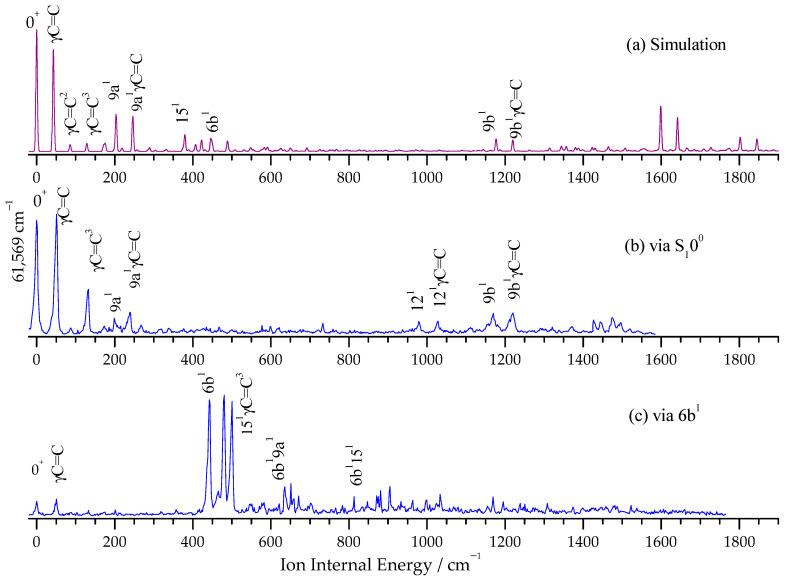
Simulated spectrum of the D_0_ ← S_1_0^0^ transition of the MAS *cis* rotamer (**a**) and its MATI spectra via the intermediate states S_1_0^0^ (**b**) and S_1_6b^1^ (**c**).

**Figure 5:**
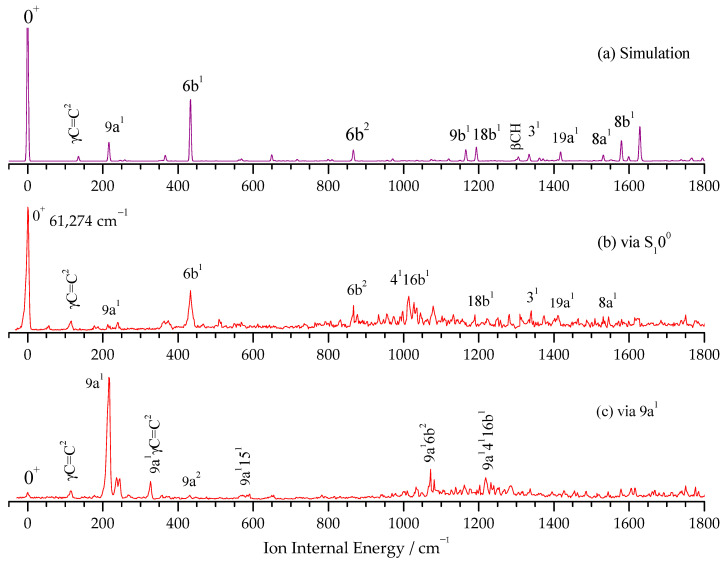
Simulated spectrum of the D_0_ ← S_1_0^0^ transition of the MAS *trans* rotamer (**a**) and its MATI spectra via the intermediate states S_1_0^0^ (**b**) and S_1_9a^1^ (**c**).

To probe the ionic properties, MATI spectra were recorded for both rotamers via their respective S_1_ origins and other intermediate vibronic levels (e.g., 6b^1^ for *cis* in Figure 4; 0^0^ and 9a^1^ for *trans* in Figure 5). Analysis of the MATI spectrum via the S_1_ 0^0^ state yields the adiabatic ionization energy (AIE). The IE for the *cis* rotamer is determined to be 61,569 ± 5 cm^−1^ and for the *trans* rotamer 61,274 ± 5 cm^−1^. The errors mainly arise from electric field effects, Rydberg state decay, laser calibration drift, the Doppler effect and external magnetic disturbances. These experimental IEs are in good agreement with the high-level theoretical calculations (see Section 3.1), further validating the rotamer assignment.

The MATI spectra also reveal the vibrational structure of the D_0_ state. The active vibrational modes for the *cis* and *trans* cations are listed in Table 3 and Table 4, respectively. The spectra are dominated by vibrations that are active in the S_1_ state. A key observation is the Δν = 0 propensity rule: when a specific vibronic level of the S_1_ state (e.g., 6b^1^) is used as the intermediate state, the most intense band in the MATI spectrum corresponds to the same vibrational level (6b^1^) in the D_0_ state. This indicates a high degree of similarity in the molecular geometry and normal coordinates between the S_1_ and D_0_ states.

Nevertheless, several distinctions between the S_1_ and D_0_ states are noteworthy. As can be seen from Table 3 and Table 4, upon D_0_ ← S_1_ ionization, out-of-plane vibrational modes of the amino group are not activated. In contrast, low-frequency (sub-60 cm^−1^) vinyl C=C torsional modes (γC=C), along with their overtones and combination bands, are extensively observed. In addition, numerous in-plane vibrational modes are activated, suggesting that the aromatic ring undergoes some in-plane structural changes during the ionization process.

## 3. Discussion

### 3.1. Excitation and Ionization Energies

**Figure 6:**
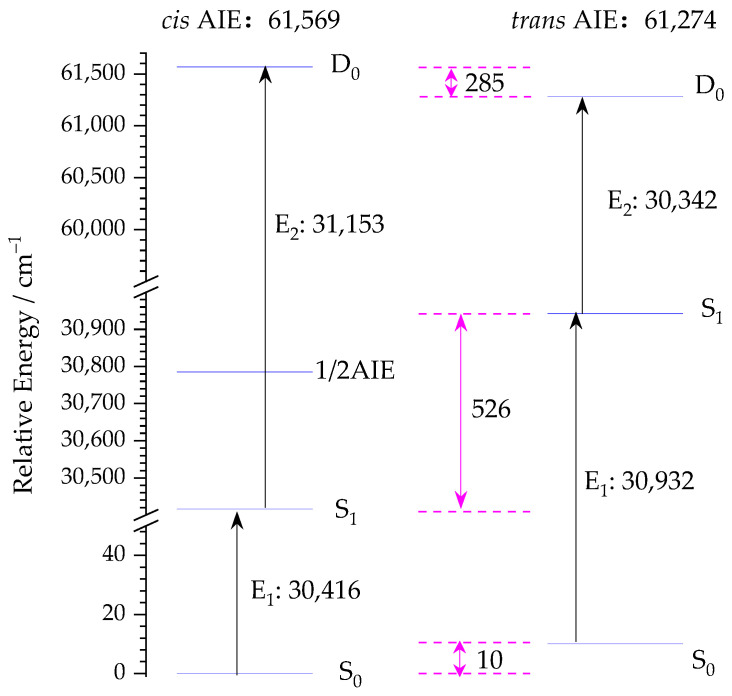
Schematic energy level diagram of *cis* and *trans* rotamers of MAS (not drawn to scale). All values are in units of cm^−1^. The energy differences in the electronic ground state were obtained from B3LYP/aug-cc-pVTZ calculations; all other energy data were taken from the present experimental measurements.

The experimentally determined S_1_ ← S_0_ (E_1_) and D_0_ ← S_1_ (E_2_) transition energies, along with the IEs, are summarized in Table 5 and illustrated in the energy level diagram Figure 6. The experimental IE for the *cis* rotamer is 61,569 cm^−1^, and for the *trans* rotamer is 61,274 cm^−1^. The small difference of 295 cm^−1^ between the two rotamers reflects the subtle influence of the orientation of the vinyl group relative to the amino group on the cationic stability.

This energetic ordering—namely, a lower S_1_ excitation energy but higher ionization energy for the *cis* rotamer compared to the *trans* rotamer—closely resembles the energy level patterns observed for Rotamers II and IV of *m*-methoxystyrene [37], as well as for Rotamers I and III. This consistency strongly supports the correctness of our rotamer assignment.

Our high-level theoretical calculations using the G4 and CBS-QB3 methods predict IE values of 61,882 cm^−1^ (*cis*) and 61,506 cm^−1^ (*trans*) for G4, and 61,563 cm^−1^ (*cis*) and 61,290 cm^−1^ (*trans*) for CBS-QB3. The CBS-QB3 results show excellent agreement with our experimental data, with relative errors of only 0.097% (*cis*) and 0.026% (*trans*), validating the computational protocol for predicting the IE of such substituted styrene systems. The observed red-shift in IEs compared to aniline (62,271 cm^−1^) [17] and styrene (68,273 cm^−1^) [18] is due to the combined electron-donating effect of the amino and vinyl groups, which stabilizes the cation.

### 3.2. Structural Changes upon Excitation and Ionization

The structures of *cis* and *trans m*-aminostyrene and their atomic labeling are shown in Figure 1. The Δν = 0 propensity observed in the MATI spectra is a direct consequence of minimal geometry change between the S_1_ and D_0_ states. This is quantitatively confirmed by the calculated structural parameters for the *cis* rotamer presented in Table S1. The most notable changes from S_0_ to S_1_ are as follows. The amino group, which tilts out of the ring plane by approximately 24° in the ground state, becomes planar in the S_1_ state. This planarization leads to the observation of numerous out-of-plane vibrational modes, particularly the wagging modes of the amino group along with their overtones and combination bands. Another notable change is the elongation of the ring C–C bonds (e.g., C1–C2 from 1.398 to 1.417 Å) and the shortening of the C3–N16 bond (from 1.395 to 1.361 Å) and the C11=C13 vinyl bond (from 1.333 to 1.375 Å). This indicates an expansion of the aromatic ring upon π → π* excitation and a strengthening of the conjugation between the substituents and the ring.

Upon ionization (D_0_ ← S_1_), the structural adjustments are comparatively small. For instance, the C3–N16 bond length further shortens to 1.336 Å, confirming the quinoidal character of the cation, but the overall ring bond lengths change only subtly. The dihedral angles show that the amino group, which is non-planar in S_0_ (dihedral angle ~23.7° for C2–C3–N16–H17), becomes nearly planar in both S_1_ and D_0_ (dihedral angles ~0°). The similarity in geometry between the planar S_1_ and planar D_0_ states is the primary reason for the observed Franck–Condon propensity.

For the *trans* rotamer, as summarized in Table S2, the C3–N16 bond shortens from 1.395 Å in S_0_ to 1.358 Å in S_1_ and further to 1.335 Å in D_0_, while the amino group becomes planar (C2–C3–N16–H17 dihedral angle changes from 24.44° in S_0_ to −0.04° in S_1_ and 0.01° in D_0_), mirroring the behavior of the *cis* rotamer. For both rotamers, S_1_ ← S_0_ excitation leads to expansion of the aromatic ring, shortening of the C–NH_2_ bond, and planarization of the amino group, whereas the structural adjustments upon ionization (D_0_ ← S_1_) are comparatively small.

### 3.3. Molecular Orbital Analysis of cis and trans m-Aminostyrene

**Figure 7:**
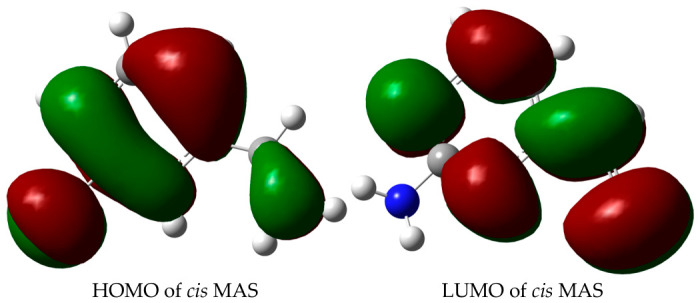

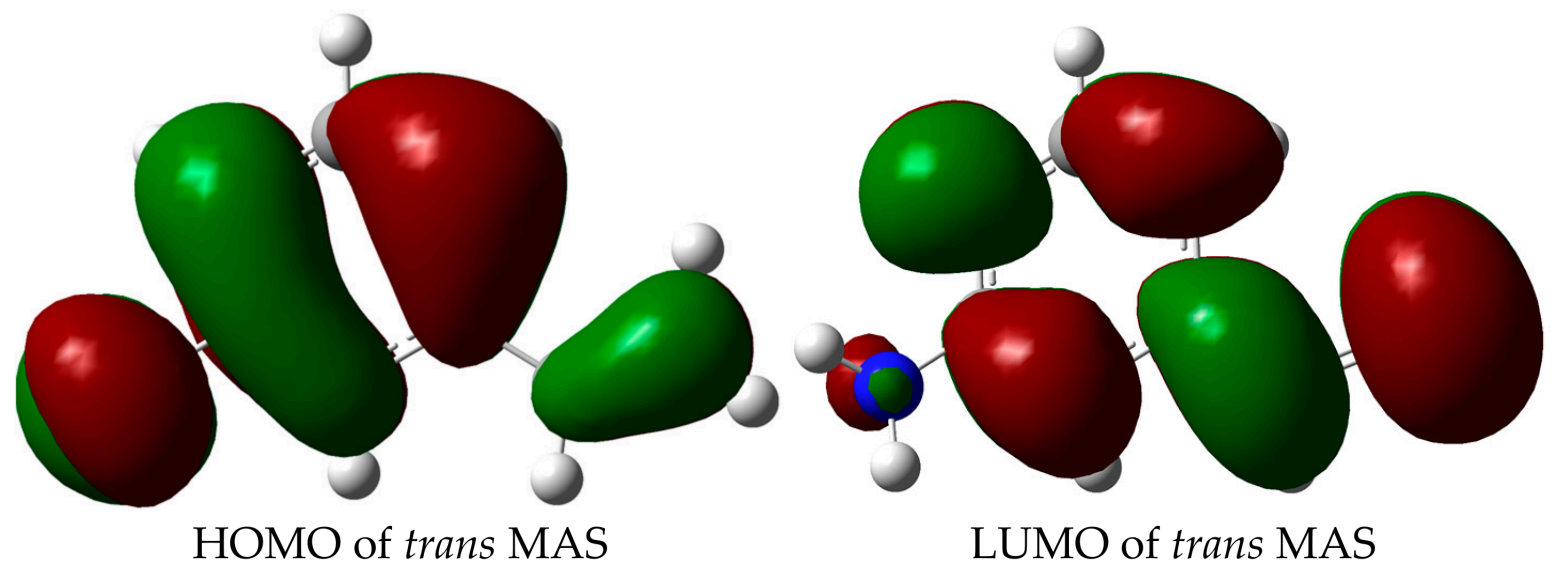
Frontier molecular orbitals (HOMO and LUMO) of *cis* and *trans m*-aminostyrene.

To gain deeper insight into the observed spectroscopic behavior, we analyzed the frontier molecular orbitals (HOMO and LUMO) of both cis and trans conformers of *m*-aminostyrene. The HOMO and LUMO orbitals were visualized from the B3LYP/aug-cc-pVTZ optimized ground-state geometries, as shown in Figure 7.

For both conformers, the HOMO is primarily localized on the aniline moiety, i.e., the amino group and the adjacent aromatic ring, with some extension toward the vinyl group. In contrast, the LUMO is mainly distributed over the aromatic ring and the vinyl group, with negligible density on the amino group. This spatial separation indicates that the S_0_ → S_1_ excitation is dominated by a π → π* transition, where electron density is transferred from the electron-donating amino group (and the ring) toward the vinyl group and the opposite side of the ring. This charge redistribution upon excitation is consistent with the observed intense bands in the REMPI/HB spectra.

Regarding the energetic ordering of the cis and trans rotamers, the slight energy difference observed both experimentally and computationally can be rationalized by subtle differences in through-space interactions. In the cis conformer, the vinyl group is oriented closer to the NH_2_ group, leading to a slightly different conjugation efficiency and a weak steric/electronic perturbation compared to the trans conformer. This conformational effect is reflected in the calculated HOMO–LUMO gaps and vertical excitation energies, which reproduce the experimental trend.

Overall, the molecular orbital analysis supports the assignment of the experimental electronic transitions and provides a clear electronic-structure basis for the observed spectroscopic differences between the two rotamers.

### 3.4. Vibrational Assignments and Substituent Effects

The comprehensive assignment of vibrational modes in Table 1, Table 2, Table 3 and Table 4 provides a detailed map of the vibronic and cationic structure of MAS, where several modes are of particular interest. The NH_2_ wagging (ω) mode, observed at 378 cm^−1^ in the *cis* S_1_ spectrum, shows frequency and intensity that are sensitive to the planarity of the amino group. The ring breathing mode (1^1^) appears at 701 cm^−1^ (*cis* S_1_) and shifts to a higher frequency of 733 cm^−1^ in the D_0_ state, reflecting the increased rigidity of the ring in the cation. Notably, the vibrational frequencies of the *cis* and *trans* rotamers are distinct, which is particularly useful for identifying and characterizing each species in a mixture, as demonstrated here.

## 4. Materials and Methods

### 4.1. Experimental Method

The experiments were performed on a home-built molecular beam time-of-flight (TOF) mass spectrometer. A detailed description is provided in our previous publications [23,57,58]. Briefly, MAS (98% purity, Shanghai Titan Technology Co., Shanghai, China) was used without further purification, and heated to ~150 °C in a nozzle reservoir to achieve sufficient vapor pressure. The vapor was seeded into 3 bar of krypton and expanded into a vacuum chamber through a pulsed valve (0.5 mm orifice) to form a supersonic molecular beam. The source and ionization chambers were maintained at pressures of 8 × 10^−4^ Pa and 3 × 10^−6^ Pa, respectively.

For the 2C-REMPI and MATI experiments, two tunable UV laser systems were used. The probe or excited laser was a Nd:YAG (Qsmart 850, Quantel, Les Ulis, Essonne, France) pumped dye laser (Precision Scan-D, Sirah GmbH, Gottingen, Germany; DCM/DMSO dye), whose output was frequency-doubled to cover 315–330 nm. The ionization laser was a separate Nd:YAG (Qsmart 850) pumped dye laser (Cobra-Stretch, Sirah GmbH, Gottingen, Germany; DCM dye), also frequency-doubled, providing a fixed wavelength at 317.96 nm for 2C-REMPI or scanned for IE measurements and MATI. For the UV-UV HB experiment, a third tunable UV laser (Precision Scan-D) was used as the “burn” laser, while the other two lasers acted as the “probe” (fixed at a resonant wavelength for a specific rotamer). The third laser beam (266 nm) is derived from the second harmonic generation of a beam splitter (approximately 8 mJ) from a YAG 532 nm laser. The three laser beams were spatially and temporally overlapped in the ionization region with a timing sequence controlled by a delay generator (DG645, Stanford Research Systems, Inc., Sunnyvale, CA, USA). In the MATI experiments, a weak spoiling electric field (0.7 V/cm) was applied to discriminate against prompt ions, followed by a pulsed field (143 V/cm) to field-ionize long-lived Rydberg states, and the resulting threshold ions were detected in the TOF mass spectrometer. Data points were averaged over 300 laser shots per wavelength, and the absolute wavelengths of both dye lasers were calibrated using a wavemeter (HighFinesse WS-7, HighFinesse GmbH, Offenburg, Germany).

### 4.2. Theoretical Calculations

All quantum chemical calculations were performed using the Gaussian 16 software package [59]. Geometry optimizations and harmonic vibrational frequency calculations for the ground state (S_0_), the first electronically excited state (S_1_), and the cationic ground state (D_0_) were carried out using density functional theory (DFT) with the B3LYP functional [15,16] and the Dunning-type aug-cc-pVTZ basis set. This basis set which includes diffuse functions, is superior to Pople-type basis sets for describing excited, Rydberg, and ionized states in spectral calculations. The excited state (S_1_) was calculated using time-dependent DFT (TD-DFT) at the same level of theory. It should be noted that the TD-B3LYP/aug-cc-pVTZ approach, while computationally efficient and widely applied to valence excited states, does not include long-range correction. This may lead to underestimated excitation energies for states with prominent charge-transfer (CT) character. Nevertheless, the S_1_ state of *m*-aminostyrene is dominated by local ππ* transitions instead of CT character, so this method yields results in reasonable agreement with experimental data. For the cationic ground state (D_0_), which is an open-shell system, the unrestricted B3LYP (UB3LYP) functional was employed. Frequency scaling factors of 0.971 (S_1_) and 0.994 (D_0_) were determined by comparing calculated and experimental frequencies to correct for anharmonicity and basis set incompleteness. Franck–Condon (FC) spectral simulations were performed to aid in the assignment of vibrational spectra. In addition, high-level ab initio calculations, specifically G4 and CBS-QB3 methods, were employed to predict the ionization energies of the two rotamers. The theoretical predictions provided valuable guidance for the execution of the experimental work and the interpretation of the experimental data.

## 5. Conclusions

In this study, we have successfully performed a comprehensive, rotamer-resolved spectroscopic investigation of *m*-aminostyrene using a combination of 2C-REMPI, UV-UV hole-burning, and MATI techniques, validated by high-level theoretical calculations.

We unambiguously separated and assigned the vibronic spectra of the *cis* and *trans* rotamers of MAS, overcoming the limitations of previous 1C-R2PI work. The adiabatic S_1_ ← S_0_ excitation energies are 30,416 cm^−1^ (*cis*) and 30,932 cm^−1^ (*trans*).

For the first time, the adiabatic IEs for both rotamers are accurately determined: 61,569 ± 5 cm^−1^ (*cis*) and 61,274 ± 5 cm^−1^ (*trans*). The calculated IEs using the CBS-QB3 method show remarkable agreement with the experimental values.

The Δν=0 propensity observed in the MATI spectra, corroborated by DFT-calculated geometries, demonstrates a very similar molecular structure between the S_1_ and D_0_ states. Both states are found to be planar with significant conjugation, whereas the S_0_ state is non-planar at the amino group.

A detailed assignment of the vibrational modes in both the S_1_ and D_0_ states is provided, serving as a valuable reference for future studies on substituted styrenes.

This work not only provides deep insights into the photophysics of a model push–pull styrene but also demonstrates a powerful methodological blueprint for studying complex molecular systems with multiple conformers.

## Figures and Tables

**Table 1:** Transition energies, relative intensities, and assignments of observed bands in the REMPI/HB experiment of cis MAS, along with calculated vibrational frequencies (in cm^−1^) ^a^.

Transition Energy	Exp.	Relative Intensity	Calc. ^a^	Assignment ^b^
30,416	0	100	0	0
30,577	161	1	165	10b^1^
30,614	198	24	190	9a^1^
30,670	254	33	263	10b^1^γC=C
30,703	287	38	290	10a^1^γC=C
30,772	356	6	358	10a^1^10b^1^
30,794	378	19	370	ωNH_2_
30,852	436	58	435	6b^1^
30,917	501	22	515	6a^1^
30,949	533	8	530	βC=C
31,117	701	13	703	1^1^
31,173	757	12	764	17b^1^
31,215	799	25	804	6b^1^ωNH_2_
31,245	829	46	833	γCH
31,275	859	1	860	17b^1^γC=C
31,370	954	17	954	12^1^
31,531	1115	8	1113	15^1^ωNH_2_^2^
31,590	1174	17	1174	6b^1^ωNH_2_^2^
31,683	1267	5	1269	ωNH_2_^2^βC=C
31,744	1328	5	1323	12^1^ωNH_2_
31,806	1390	3	1391	19a^1^
31,872	1456	6	1459	9a^1^ωNH_2_^2^βC=C

^a^ The experimental values are shifts from 30,416 cm^−1^, whereas the predicted values are obtained from the B3LYP/aug-cc-pVTZ calculations, scaled by 0.971. ^b^ Internal vibrations of the substituents: β, in-plane bending; γ, out-of-plane bending; ω, wagging of the amino group.

**Table 2:** Transition energies, relative intensities, and assignments of observed bands in the REMPI/HB experiment of *trans* MAS, along with calculated vibrational frequencies (in cm^−1^) ^a^.

Transition Energy	Exp.	Relative Intensity	Calc. ^a^	Assignment ^b^
30,932	0	100	0	0
31,082	150	3	142	10a^1^
31,136	204	66	206	9a^1^
31,204	272	41	279	10a^2^
31,265	333	1	337	16b^1^
31,296	364	2	356	ωNH_2_
31,340	408	2	411	9a^2^
31,361	429	6	428	6b^1^
31,422	490	11	508	6a^1^
31,565	633	7	634	6b^1^9a^1^
31,627	695	9	697	6a^1^γC=C^2^
31,717	785	2	784	6b^1^ωNH_2_
31,753	821	7	813	16a^1^ωNH_2_
31,852	920	2	917	9a^1^ωNH_2_^2^
31,887	955	3	955	12^1^
31,913	981	1	989	6b^1^ωNH_2_^1^9a^1^

^a^ The experimental values are shifts from 30,932 cm^−1^, whereas the predicted values are obtained from the B3LYP/aug-cc-pVTZ calculations, scaled by 0.979. ^b^ Internal vibrations of the substituents: γ, out-of-plane bending; ω, wagging of the amino group.

**Table 3:** Vibrational frequencies (in cm^−1^) and assignments of observed bands in the MATI experiment of *cis* MAS ^a^.

Intermediate Levels in the S_1_ State		Calc. ^a^	Assignment ^b^
0^0^	6b^1^		
51	51	43	γC=C
88	87	85	γC=C^2^
133	133	129	γC=C^3^
174	175	175	10b^1^
199	201	203	9a^1^
240		246	9a^1^γC=C
268		257	γC=C^6^
377		380	15^1^
435		432	16b^1^γC=C
445	442	446	6b^1^
	481	488	6b^1^γC=C
	500	508	15^1^γC=C^3^
577		583	9a^1^15^1^
599		591	βC=CγC=C
621		626	9a^1^15^1^γC=C
	635		6b^1^9a^1^
	651		6b^1^9a^1^γC=C
733		725	1^1^
	813		6b^1^15^1^
979		988	12^1^
1026	1033	1030	12^1^γC=C
1170	1169	1177	9b^1^
1220		1220	9b^1^γC=C

^a^ The experimental values are shifts from 61,569 cm^−1^, whereas the predicted values are obtained from UB3LYP/aug-cc-pVTZ calculations, scaled by 0.994. ^b^ Internal vibrations of the substituents: β, in-plane bending; γ, out-of-plane bending.

**Table 4:** Vibrational frequencies (in cm^−1^) and assignments of observed bands in the MATI experiment of *trans* MAS ^a^.

Intermediate Levels in the S_1_ State		Calc. ^a^	Assignment ^b^
0^0^	9a^1^		
57		68	γC=C
116	115	135	γC=C^2^
213	216	216	9a^1^
239	240		10a^1^γC=C
	327		9a^1^γC=C^2^
363		366	15^1^
375		370	10a^1^10b^1^
	431		9a^2^
433		433	6b^1^
509		501	15^1^γC=C^2^
	572		9a^1^15^1^
	649		9a^1^6b^1^
867		867	6b^2^
933		933	1^1^9a^1^
955		956	16a^2^
973		971	12^1^
1014		1014	4^1^16b^1^
1035		1036	βNH_2_
	1072		9a^1^6b^2^
1079		1082	6b^2^9a^1^
1190		1194	18b^1^
1309		1305	βCH
1340		1334	3^1^
1532		1531	8a^1^

^a^ The experimental values are shifts from 61,274 cm^−1^, whereas the predicted values are obtained from UB3LYP/aug-cc-pVTZ calculations, scaled by 0.994. ^b^ Internal vibrations of the substituents: β, in-plane bending; γ, out-of-plane bending.

**Table 5:** Theoretical and experimental adiabatic ionization energies of *cis* and *trans* MAS, and their relative errors.

	*cis*	*trans*
Experimental IE (cm^−1^)	61,569	61,274
Calculated IE (G4, cm^−1^)	61,882	61,506
Relative error	0.51%	0.38%
Calculated IE (CBS-QB3, cm^−1^)	61,563	61,290
Relative error	0.097%	0.026%

## Data Availability

The data that support the findings of this study are available from the corresponding author, Changyong Li, upon reasonable request.

## Supplementary Materials

The following supporting information can be downloaded at: https://www.mdpi.com/article/10.3390/molecules31111866/s1. Table S1: Geometric parameters of the *cis* rotamer of MAS in the S_0_, S_1_, and D_0_ states calculated at B3LYP/aug-cc-pVTZ, TD-B3LYP/aug-cc-pVTZ, and UB3LYP/aug-cc-pVTZ levels, respectively. Table S2: Geometric parameters of the *trans* rotamer of MAS in the S_0_, S_1_, and D_0_ states calculated at B3LYP/aug-cc-pVTZ, TD-B3LYP/aug-cc-pVTZ, and UB3LYP/aug-cc-pVTZ levels, respectively.
