# Supplementary material for: Rotamer-Resolved Vibronic and Cationic Properties of m-Aminostyrene: A Combined 2C-REMPI, Hole-Burning, and MATI Study
Source: Molecules. 2026 May 29;31(11):1866. doi: 10.3390/molecules31111866 (PMC13258208; doi:10.3390/molecules31111866)
Supplement: Supplementary file 1 [file molecules-31-01866-s001.zip › molecules-4343602-supplementary.pdf]

Supporting Information

Rotamer-Resolved Vibronic and Cationic Properties of m-Aminostyrene: A Combined 2C-REMPI, Hole-Burning, and MATI Study

Rui Wang [1], Xiateng Qin [1], Keke Zhang [1], Yan Zhao[2,*], Changyong Li [1,3,*] and Suotang Jia [1,3,*]

1 State Key Laboratory of Quantum Optics Technologies and Devices, Institute of Laser Spectroscopy, Shanxi University, Taiyuan 030006, China; 19861241857@163.com (R.W.); 18734558738@163.com (X.Q.); zhangkeke323@163.com (K.Z.);

2 Department of Physics and Electronics Engineering, Jinzhong University, Jinzhong 030619, China;

3 Collaborative Innovation Center of Extreme Optics, Shanxi University, Taiyuan 030006, China

* Correspondence: zhaoy@jzxy.edu.cn (Y.Z.); lichyong@sxu.edu.cn (C.L.); tjia@sxu.edu.cn (S.J.)

Table S1. Geometric parameters of the *cis* rotamer of MAS in the S_0, S_1, and D_0 states calculated at B3LYP/aug-cc-pVTZ, TD-B3LYP/aug-cc-pVTZ, and UB3LYP/aug-cc-pVTZ levels, respectively.

	S_0	S_1	D_0	**Δ(S_1 − S_0)**	**Δ(D_0 − S_1)**
Bond length (Å)					
C1–C2	1.398	1.417	1.379	0.019	-0.038
C2–C3	1.394	1.413	1.413	0.019	-0.001
C3–C4	1.402	1.412	1.437	0.011	0.025
C4–C5	1.385	1.393	1.369	0.008	-0.024
C5–C6	1.389	1.394	1.399	0.005	0.004
C6–C1	1.399	1.431	1.432	0.032	0.001
C1–C11	1.469	1.426	1.460	-0.044	0.034
C11–C13	1.333	1.375	1.336	0.042	-0.039
C3–N16	1.395	1.361	1.336	-0.035	-0.025
Bond angle (°)					
C1–C2–C3	121.42	121.00	120.40	-0.42	-0.61
C2–C3–C4	118.94	123.00	120.41	4.06	-2.59
C3–C4–C5	120.00	116.33	119.33	-3.68	3.00
C4–C5–C6	120.75	121.20	119.74	0.44	-1.46
C5–C6–C1	120.20	123.82	122.08	3.62	-1.74
C6–C1–C2	118.68	114.66	118.05	-4.02	3.39
C2–C1–C11	122.72	123.50	124.39	0.78	0.89
C6–C1–C11	118.59	121.84	117.56	3.24	-4.28
C1–C11–C13	127.84	126.32	126.18	-1.53	-0.14
C2–C3–N16	120.67	117.95	120.86	-2.72	2.91
C4–C3–N16	120.33	119.06	118.74	-1.28	-0.32
C3–N16–H17	115.75	120.87	121.51	5.13	0.64
C3–N16–H18	115.68	120.99	121.48	5.31	0.50
Dihedral angle					
C2–C3–N16–H17	23.74	-0.02	0.18	-23.76	0.19
C2–C3–N16–H18	158.00	180.00	-179.71	22.00	0.29
C4–C3–N16–H17	-159.03	179.99	179.88	-20.98	-0.11
C4–C3–N16–H18	-24.77	0.00	-0.02	24.77	-0.01

Table S2. Geometric parameters of the *trans* rotamer of MAS in the S_0, S_1, and D_0 states calculated at B3LYP/aug-cc-pVTZ, TD-B3LYP/aug-cc-pVTZ, and UB3LYP/aug-cc-pVTZ levels, respectively.

	S_0	S_1	D_0	**Δ(S_1 – S_0)**	**Δ(D_0 – S_1)**
Bond length (Å)					
C1–C2	1.396	1.409	1.379	0.013	-0.031
C2–C3	1.398	1.407	1.413	0.009	0.006
C3–C4	1.397	1.422	1.435	0.025	0.013
C4–C5	1.390	1.386	1.372	-0.004	-0.013
C5–C6	1.386	1.393	1.396	0.008	0.002
C6–C1	1.401	1.446	1.434	0.045	-0.013
C1–C11	1.469	1.420	1.456	-0.049	0.035
C11–C13	1.333	1.383	1.338	0.051	-0.045
C3–N16	1.395	1.358	1.335	-0.037	-0.022
Bond angle (°)					
C1–C2–C3	121.83	121.54	120.69	-0.29	-0.85
C2–C3–C4	118.56	122.61	120.00	4.05	-2.61
C3–C4–C5	119.95	116.56	119.43	-3.39	2.87
C4–C5–C6	121.23	121.37	120.09	0.15	-1.29
C5–C6–C1	119.79	123.39	121.60	3.60	-1.79
C6–C1–C2	118.64	114.53	118.20	-4.11	3.67
C2–C1–C11	118.27	123.10	120.00	4.83	-3.10
C6–C1–C11	123.09	122.37	121.80	-0.71	-0.57
C1–C11–C13	127.56	126.09	126.22	-1.47	0.13
C2–C3–N16	120.53	119.44	121.02	-1.09	1.58
C4–C3–N16	120.86	117.95	118.98	-2.91	1.03
C3–N16–H17	115.76	121.14	121.46	5.38	0.33
C3–N16–H18	115.68	120.80	121.51	5.11	0.71
Dihedral angle					
C2–C3–N16–H17	24.44	-0.04	0.01	-24.48	0.05
C2–C3–N16–H18	158.74	180.03	179.99	21.29	-0.04
C4–C3–N16–H17	-158.41	-180.03	-179.98	-21.62	0.05
C4–C3–N16–H18	-24.11	0.03	0.00	24.14	-0.03
